# Awareness, Attitudes, and Willingness: A Cross-Sectional Study of Organ Donation in Saudi Arabia

**DOI:** 10.3390/healthcare11243126

**Published:** 2023-12-08

**Authors:** Khalid Alhasan, Fadi Aljamaan, Aziza Ajlan, Hassan Aleid, Talal Al Ghoufi, Saleh I. Alabbad, Rezqah F. AlDhaferi, Weiam Almaiman, Tariq Ali, Alaa Abdullah Hakami, Rafeef Abdullah Hakami, Baraah S. Alqarni, Alhanouf S. Alrashed, Tarfa R. Alsharidi, Hamad A. Almousa, Ibraheem Altamimi, Ali Alhaboob, Amr Jamal, Mohamed A. Shalaby, Jameela A. Kari, Rupesh Raina, Dieter C. Broering, Mohamad-Hani Temsah

**Affiliations:** 1 College of Medicine, King Saud University, Riyadh 11362, Saudi Arabia; 2 Kidney and Pancreas Health Center, Organ Transplant Center of Excellence, King Faisal Specialist Hospital and Research Center, Riyadh 11564, Saudi Arabia; 3 Pediatric Department, King Saud University Medical City, King Saud University, Riyadh 11362, Saudi Arabia; 4 Critical Care Department, King Saud University Medical City, King Saud University, Riyadh 11362, Saudi Arabia; 5 Transplant Clinical Pharmacy Section, Organ Transplant Center of Excellence, King Faisal Specialist Hospital and Research Center, Riyadh 11564, Saudi Arabia; 6 Saudi Center of Organ Transplantation, Riyadh 12823, Saudi Arabia; 7 King Fahd Central Hospital, Jazan 82666, Saudi Arabia; 8 Faculty of Medicine, Jazan University, Jazan 82911, Saudi Arabia; 9 Family and Community Medicine Department, King Saud University, Riyadh 11362, Saudi Arabia; 10 Evidence-Based Healthcare and Knowledge Translation Research Chair, King Saud University, Riyadh 11421, Saudi Arabia; 11 Pediatric Nephrology Unit, Faculty of Medicine and Pediatric Nephrology Center of Excellence, King Abdulaziz University Hospital, King Abdulaziz University, Jeddah 21589, Saudi Arabia; 12 Akron Nephrology Associates, Department of Nephrology, Cleveland Clinic Akron General Medical Center, Akron, OH 44302, USA

**Keywords:** organ donation, Saudi Arabia, health surveys, knowledge, attitudes, practice, mobile applications

## Abstract

Background: Organ transplantation is inherently dependent on the availability of organ donors. There is a noticeable paucity of literature addressing the rates of organ donation registration and the awareness of Islamic regulations (Fatwa) regarding organ donation within Saudi Arabia. Our study aimed to evaluate the level of organ donation registration, awareness of Islamic regulations, and knowledge of the Saudi Center for Organ Transplantation (SCOT) within the Saudi society. Methods: We conducted a cross-sectional survey from 30 March to 9 April 2023. This survey aimed to assess the awareness of Islamic (Fatwa) guidance on organ donation, the role of SCOT, and the rate of organ donation registration facilitated through the Tawakkalna app, the official health passport application in Saudi Arabia. Results: Out of 2329 respondents, 21% had registered as potential deceased organ donors, despite 87% acknowledging the importance of organ donation. Awareness of the Islamic Fatwa regarding organ donation was reported by 54.7% of respondents, and 37% recognized the Fatwa’s acceptance of brain death criteria. The likelihood of registration as organ donors was higher among Saudi citizens under 45 years of age, females, healthcare workers (HCWs), individuals with higher education, relatives of patients awaiting organ donations, those informed about the Islamic Fatwas, and those willing to donate organs to friends. Conversely, being over the age of 25, Saudi nationality, employment as an HCW, awareness of SCOT, and prior organ donation registration were predictive of a heightened awareness of Islamic Fatwas. However, perceiving the importance of organ donation correlated with a lower awareness of the Fatwas. Significant positive correlations were found between awareness of SCOT, awareness of Fatwas, and registration for organ donation. Conclusions: While the Saudi population exhibits a high regard for the importance of organ donation, this recognition is not adequately translated into registration rates. The discrepancy may be attributable to limited awareness of SCOT and the relevant Islamic Fatwas. It is imperative to initiate organ donation awareness campaigns that focus on religious authorization to boost organ donation rates and rectify prevalent misconceptions.

## 1. Introduction

Organ transplantation represents a fundamental life-saving measure for patients with end-stage organ failure and is universally acclaimed as a substantially effective treatment for renal replacement in cases of kidney failure [1]. Despite advancements in medicine, such as the introduction of the hepatitis B vaccine and innovative treatments for hepatitis C, the prevalence of chronic liver disease continues to rise [2]. This increase is compounded by the growing incidence of nonalcoholic fatty liver disease, which is expected to surge in parallel with the rising rates of diabetes mellitus and drug-induced liver injury [2,3]. Consequently, these escalating rates of chronic liver conditions have heightened the demand for liver transplants. However, the donor pool remains insufficient to meet the current and anticipated needs, often resulting in the deaths of patients on the waiting list due to donor scarcity [4]. Organ donation can substantially improve the quality of life and decrease the mortality rate for these individuals. Globally, the demand for organ transplantation far exceeds the supply, leading to a significant disparity between the number of patients on waiting lists and the available organs for transplantation [5,6,7].

The inception of organ transplantation in the Kingdom of Saudi Arabia (KSA) dates to 1979, with the first kidney transplant from a living donor [8]. The Saudi Center for Organ Transplantation (SCOT), established in 1985, serves as the governmental body in charge of managing all transplant-related activities within the KSA. Since its establishment, organ transplantation has made considerable strides in the Kingdom. SCOT is central to various transplant-related endeavors, including educational outreach, allocation protocols, coordination, and organ procurement [8]. However, the rates of deceased organ donation remain markedly low. An individual’s willingness to donate organs is shaped by their knowledge and personal convictions about organ donation. In the Saudi context, religious misconceptions can profoundly affect decision making, particularly concerning deceased donations [9]. Health literacy also plays a crucial role in influencing organ donation decisions [10]. Initiatives to enhance education on this subject in schools and hospitals are deemed essential to increasing the pool of potential donors [11]. Additionally, social media has become a formidable platform for raising public awareness about organ donation [12].

In Saudi Arabia, despite ongoing research, there has been a lack of investigation into the actual public registration rates for potential deceased organ donors. The predominantly Muslim Saudi society’s attitudes towards organ donation are significantly shaped by Islamic jurisprudence, which also governs the legal considerations of brain death and organ transplantation. The definition of death in Islam and the legitimacy of organ harvesting at that stage remain a topic of debate [13]. Our study examines the influence of the widely recognized Islamic Fatwa issued by the Council of Islamic Jurisprudence in 1986 [14,15], seeking to evaluate the actual registration of the Saudi population as potential deceased organ donors. We also aim to explore their motivations and barriers to registration, their awareness of the Islamic Jurist (Fatwa) concerning brain death and organ donation, and their knowledge of the Saudi Council for Organ Transplantation.

## 2. Methods

### 2.1. Study Design

We conducted a cross-sectional survey from 30 March to 9 April 2023, to establish the rate of registration among the Saudi population as potential deceased organ donors. The survey also sought to evaluate awareness regarding the importance of organ donation and the Islamic Fatwas concerning brain death and organ donation, as 93% of the population in Saudi Arabia are Muslim [16].

### 2.2. Sampling, Participants and Data Collection

We disseminated survey invitations via social media platforms, using the snowball technique to achieve our sample with convenience sampling methods such as WhatsApp, Facebook, and Twitter. Participants were required to give informed consent electronically before engaging in the survey. Adults aged 18 and over residing in Saudi Arabia during the study period were eligible for inclusion. The survey platform guaranteed the anonymity of respondents and the confidentiality of their data. Monitoring of data collection was conducted daily, and the survey concluded once the target sample size was achieved.

### 2.3. The Survey Instrument

A structured questionnaire, compiled after a thorough literature review, was employed and validated for face and content validity by a panel of experts, including transplantation consultants, organ donation program directors, transplantation pharmacists, and intensivists in both adult and pediatric care.

The questionnaire, detailed in Appendix A, included segments on demographic data, registration as potential deceased organ donors via the Tawakkalna app [17], perceptions of the importance of organ donation, incentives and barriers to organ donation registration, and awareness of the Islamic Fatwas regarding brain death, organ donation, and the role of SCOT.

### 2.4. Sample Calculation and Recruitment

Based on the presumption that 50% of the population would consent to organ donation, we calculated a required sample size of 348 participants to determine the true proportion with a 95% confidence interval and a 5% margin of error. Nevertheless, the survey remained open for additional weeks, yielding a final sample size of 2329 respondents.

### 2.5. Statistical Data Analysis

Categorical variables were described using frequencies and percentages. As all variables were categorical, no symmetry analysis was applied. Multiple-response dichotomy analysis was utilized for questions with multiple responses. Multivariable logistic binary regression analysis (MLBR) was conducted to identify statistically significant predictors of organ donation registration, awareness of Islamic organ donation regulations, and awareness of SCOT among participants. The outcomes of MLBR were reported as odds ratios (OR) with 95% confidence intervals. Statistical analysis was performed using the IBM SPSS software, version 21. An alpha significance level was set at 0.050.

### 2.6. Ethical Considerations

Prior to data collection, ethical approval was granted by the Institutional Review Board (IRB) at King Saud University in Riyadh, Saudi Arabia. Study objectives were clearly communicated to participants, who provided voluntary electronic informed consent. Participants retained the right to withdraw at any stage without consequence. All data were securely stored to maintain confidentiality throughout the study.

## 3. Results

The survey was completed by 2329 participants, with a descriptive analysis of their demographics presented in Table 1. The gender distribution was nearly equal, and participants were evenly spread across the age groups of 18–24, 25–34, and 35–44 years, with those aged 45–54 years constituting 15.8% and 55–64 years at 7.1%. The majority of the participants (87.6%) were Saudi nationals, while the remaining 12.4% comprised non-Saudi participants, including both Arabs and non-Arabs. Educational levels varied: half held a bachelor’s degree, almost a quarter had completed high school, 10.4% possessed a master’s degree, and 12.7% had earned a doctorate. Healthcare workers (HCWs) made up 44.9% of the sample, with the remaining 55.1% being non-HCWs.

**Figure 1:**
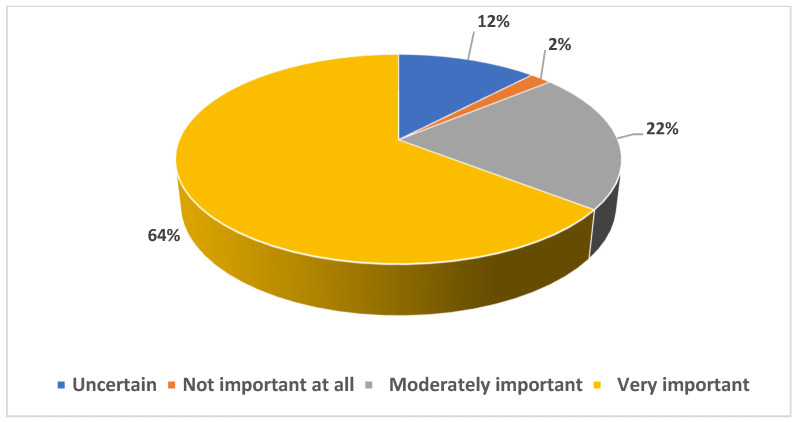
Participants’ perception of organ donation importance.

Regarding perceptions of organ donation importance, 86% viewed it as very or moderately important. A minority, 12%, remained uncertain, and a mere 2% deemed it unimportant (see Figure 1).

**Figure 2:**
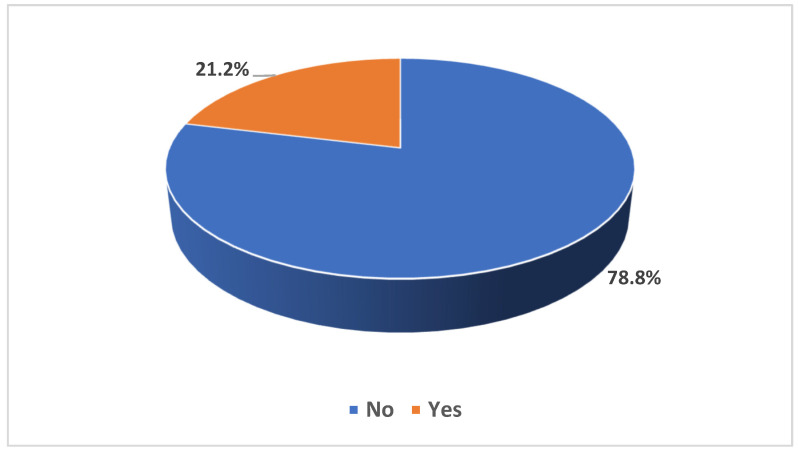
Participants registered as potential deceased organ donors, based on registration in governmental health passport (Tawakkalna app).

When considering registration as potential organ donors via the Tawakkalna app, 78.8% had not registered, contrasting with 21.2% who had (refer to Figure 2). Of those registered, a substantial 89.2% were willing to donate all organs. The remainder were willing to donate specific organs, including kidneys (9.5%) and livers (7.7%, as detailed in Table A1).

**Figure 3:**
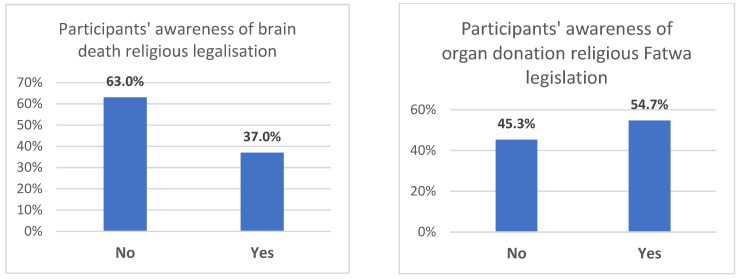
Participants’ awareness of brain death and organ donation religious Fatwas (legalization).

Belief in the concept of brain death was affirmed by 93.6% of respondents, but only 37% were aware of the Islamic Fatwa related to brain death, and 54.7% knew of the Fatwa concerning organ donation (as indicated in Figure 3). The motives and barriers influencing organ donation registration are elucidated in Table 2. Among registrants, the primary motive (90.9%) was personal or religious conviction in the value of saving lives. Additionally, 53.7% cited national donor shortages, and 52.5% aimed to leave a positive legacy. Some (21.4%) considered it prudent in case they themselves needed a transplant, while 15.7% had personal connections to someone in need of a transplant.

For non-registrants, barriers included skepticism about organ donation (37.7%), lack of time (22.7%), and lack of awareness (22.2%). Other reasons (17.4%) encompassed requiring more time for decision making (17.6%), religious uncertainties (11.9%), perceived personal unsuitability due to comorbidities or age (12.9%), anticipated family disapproval (15.3%), and concerns about the post-mortem treatment of their body (7.1%). Participants also offered suggestions for enhancing organ donation awareness and registration rates. The most endorsed strategy was increasing societal awareness about the Fatwa supporting organ donation (66.5%), followed by intensifying awareness campaigns (63.1%, as shown in Table 2).

Table 3 provides an analysis of participant characteristics that independently predict self-registration for organ donation. Gender emerged as a significant factor, with males being less likely to register than females (OR 0.658, *p*-value = 0.001). Age also played a role; individuals aged 45 years or older were less likely to register (OR 0.894, *p*-value = 0.031). Nationality was another predictor, with Saudi citizens being significantly more likely to register compared to expatriates, showing more than twice the likelihood (OR 2.33, *p*-value < 0.001). Understanding the Islamic Fatwa regarding organ donation was a strong predictor of registration, with participants who were aware of the Fatwa being 1.810 times more likely to register (*p*-value < 0.001). Previous blood donors and healthcare workers (HCWs) were also more likely to register for organ donation (OR 1.438, *p*-value = 0.004, and OR 1.18, *p*-value = 0.013, respectively). Educational level was significant; those with university degrees were 1.214 times more likely to register (*p*-value = 0.005). Family ties to organ donation also influenced registration. Participants with a relative waiting for an organ transplant and those willing to donate organs to friends were significantly more likely to register (OR 1.46, *p*-value = 0.009, and OR 2.49, *p*-value < 0.001, respectively). Interestingly, participants who favored linking the driving license application to organ donation decisions were more inclined to register (OR 2.208, *p*-value < 0.001). Participants’ belief in the effectiveness of awareness campaigns and social media initiatives to raise organ donation registration correlated positively with higher registration rates. Conversely, awareness of the role of the Saudi Center for Organ Transplantation (SCOT) had a negative association with self-registration for organ donation. 

Table 4 provides insights into the factors that influence participants’ awareness of the Islamic Fatwa on brain death, a critical factor for understanding organ donation within an Islamic context. Participants who were aware of the role of the Saudi Center for Organ Transplantation (SCOT) were the most likely to be knowledgeable about the Fatwa, with over two and a half times greater awareness (OR 2.564, *p*-value < 0.001). A belief in clinical brain death was also a significant predictor of Fatwa awareness (OR 1.883, *p*-value = 0.004).

Registration in Tawakkalna, the official governmental health passport app, was another significant predictor, with registrants being 1.697 times more aware of the Fatwa (*p*-value < 0.001). Healthcare workers (HCWs) and those who had relatives waiting for an organ transplant were significantly more aware of the Fatwa (OR 1.652, *p*-value < 0.001, and OR 1.506, *p*-value = 0.002, respectively). Previous blood donors and individuals who supported linking driving license applications with organ donation decisions were also more likely to be aware of the Fatwa (OR 1.323, *p*-value = 0.012, and OR 1.260, *p*-value = 0.035, respectively). Age was a factor, with participants aged 25 and older being more aware of the Fatwa (OR 1.325, *p*-value < 0.001). Similarly, Saudi citizens had higher awareness than expatriates (OR 1.49, *p*-value = 0.004).

Conversely, there was an interesting inverse relationship for those who perceived organ donation as important; they were significantly less aware of the Fatwa regarding brain death (20.8% less likely, *p*-value < 0.001). This suggests that even among those who value organ donation, awareness of religious rulings related to brain death is not as prevalent.

Table 5 details the factors that contribute to participants’ awareness of the Fatwa specific to organ donation. Understanding these factors is crucial as they influence societal attitudes toward organ donation in a region where religious edicts play a significant role in guiding public opinion and behaviors. Awareness of the role played by the Saudi Center for Organ Transplantation (SCOT) remained the strongest predictor of participants being informed about the organ donation Fatwa, with an odds ratio of 2.535 (*p*-value < 0.001). This suggests that SCOT’s visibility and educational outreach are central to disseminating knowledge about religious endorsements of organ donation. Registration on Tawakkalna as a potential organ donor was also a significant factor, with registrants nearly twice as likely to be aware of the Fatwa compared to non-registrants (OR 1.916, *p* < 0.001). The pattern of increased awareness among those who supported linking the driving license process with organ donation decisions continued, with these individuals having significantly higher awareness (OR 1.265, *p* = 0.027). Similarly, individuals who had donated blood before had higher awareness levels (OR 1.329, *p* = 0.005). Both Saudi citizens and participants aged 25 and older exhibited significantly greater awareness of the Fatwa. Healthcare workers (HCWs) and individuals with relatives awaiting organ transplants also showed significantly higher awareness (OR 1.282, *p* < 0.001, and OR 1.336, *p* = 0.024, respectively). In a consistent trend with the awareness of the brain death Fatwa, individuals who held a strong belief in the importance of organ donation were paradoxically less aware of the organ donation Fatwa (22.4% times less likely, *p*-value < 0.001).

Table 6 provides insights into the personal characteristics that affect an individual’s propensity to donate organs to friends, which is an important aspect of understanding the broader attitudes toward organ donation within the society. Older participants (those aged 35 years or older) and those with higher educational attainment (master’s degree or above) were less inclined to donate organs to friends, as shown by their odds ratios of 0.920 (*p* = 0.034) and 0.769 (*p* < 0.001), respectively. This could suggest that age and education may influence personal decisions about organ donation, particularly when the potential recipients are friends rather than family members. Contrarywise, individuals who expressed a willingness to donate organs to family members were far more likely to extend that willingness to friends, with a very high odds ratio of 16.790 (*p* < 0.001). This indicates a strong correlation between one’s attitude towards donating to relatives and to friends, perhaps reflecting a broader altruistic disposition or a less strict delineation between family and friends in the context of organ donation.

Participants’ awareness of the Saudi Center for Organ Transplantation (SCOT) is an important factor in increasing organ donation awareness. Table 7 examines the characteristics associated with participants’ awareness of SCOT’s role in organ donation. The strongest predictor of this awareness was the participants’ strong belief in the importance of organ donation, with those holding this belief being more than three times as likely to be aware of SCOT (OR 3.101, *p*-value < 0.001). Awareness of the Islamic Fatwas on brain death and organ donation was also significantly associated with awareness of SCOT’s role. Those informed about the brain death Fatwa were 1.71 times more likely, and those aware of the organ donation Fatwa were 1.959 times more likely to be aware of SCOT’s role (*p*-value < 0.001 for both). The belief in the effectiveness of social media campaigns in enhancing organ donation awareness was linked with a higher awareness of SCOT’s role (OR 1.336, *p*-value = 0.007). Age and nationality were significant factors; participants aged 25 years or older and Saudi citizens were more aware of SCOT’s role (OR 1.16, *p*-value = 0.001 and OR 1.834, *p*-value < 0.001, respectively). The level of education was also a significant correlate; those holding a university degree or higher were more aware of SCOT’s role (OR 1.156, *p*-value = 0.032). As anticipated, healthcare workers had a higher awareness of SCOT’s role (OR 1.243, *p*-value < 0.001).

## 4. Discussion

Solid organ transplantation is considered the definitive treatment for end-stage organ failure, and organ donation is the critical resource for such transplantation programs. Despite this, the shortage of donor organs remains a significant barrier, with the need greatly surpassing supply [18]. The decision to donate organs can be complex, particularly in religious societies like Saudi Arabia, where theological concerns can influence attitudes towards organ donation. Islamic scholars worldwide are re-evaluating the nuances of organ donation and transplantation, an emotionally charged issue that requires updated scholarly interpretations while explicitly prohibiting commercial organ donation in Islam. Our study identifies several factors that shape the perspectives of the Saudi population on organ donation. Although religious teachings are influential, other contextual factors add to the complexity of decision making in this area.

In our research, only about one-quarter of the participants were registered as potential deceased organ donors. A study from the Kingdom of Saudi Arabia (KSA) indicated that the main reasons for not donating were misconceptions about the sufficiency of one kidney and concerns about the health of the remaining kidney [19,20]. However, a significant majority of our participants considered organ donation important. This belief was significantly associated with awareness of the Islamic Fatwa on brain death and organ transplantation, as well as knowledge of SCOT’s role, but it did not necessarily lead to registration as donors or the inclination to donate organs to friends or family members. Interestingly, the majority of those registered as deceased donors were willing to donate all potential organs, contrasting with the other literature that identifies the kidney as the most frequently donated organ [9,10,21,22,23,24,25]. This demonstrates a remarkable level of altruism within the Saudi population.

The majority of Saudis, being Muslims, adhere to scholarly Fatwas regarding the legitimacy of brain death and organ donation since these issues are deeply rooted in religious beliefs within Muslim communities. The International Islamic Jurist Council legally recognized brain death in Islam in October 1986. Subsequent Fatwas by the International Islamic Fiqh Academy (IIFA) and the Islamic Fiqh Academy (IFA) in 1990 and 2003 have supported organ transplantation, providing a solid foundation for transplant programs and ending a long-standing debate [26]. Research indicates that many Muslims still believe that both living and deceased organ donation may be forbidden in Islam, an opinion that negatively impacts their attitudes toward organ donation and transplantation [27,28].

In our study, 93% of participants believed in the concept of brain death, but only 54.7% were aware of the Fatwa on organ donation, and 37% understood the Fatwa on brain death. Awareness of organ donation was linked with a greater inclination to register as a potential donor, consistent with previous studies [29]. A comprehensive understanding of brain death can alleviate fears about being declared dead prematurely, thereby increasing the likelihood of organ donation after death [30,31]. The main issue with public awareness seems to be the insufficient dissemination and clarification of Islamic teachings on organ donation and transplantation by official bodies and Muslim scholars [32,33,34,35,36].

Muslims generally possess a strong sense of charity and altruism, which fosters a positive attitude toward organ donation, particularly for family members or friends [37,38,39,40]. In Islamic culture, organ donation is considered a noble deed, with donors expecting to gain God’s favor and receive rewards in the afterlife [38,41]. This is supported by a significant correlation between the willingness to donate to family and friends observed in our study. A clear connection has been found between understanding Islamic views on transplantation and the willingness to donate organs, either in life or after death, in Saudi Arabia. Notably, the majority of individuals in Saudi Arabia who have registered as organ donors cite religious convictions as their primary motivation [39]. In another study within the same region, willingness to donate was largely attributed to religious beliefs, while financial incentives did not significantly increase the willingness to donate [19]. The reluctance to engage in organ donation in some Muslim communities may partly stem from insufficient awareness or misunderstandings regarding the organ donation Fatwa [37].

Islamic communities often value strong family ties [42], leading Muslims to have concerns about the impact of illness or organ donation on their families. Additionally, fears that organ donation after brain death might interfere with the mourning process or cause emotional distress to the family are significant deterrents [36,38].

The Saudi Center for Organ Transplantation (SCOT) was established in 1984 to adopt strategies for organ donation, including research, public awareness, education for healthcare professionals, and collaboration with organizations supporting organ failure patients [43]. Awareness of SCOT’s role in organ transplantation is linked with understanding brain death and organ donation Fatwas. However, this awareness is inversely related to registration as potential deceased organ donors in our population.

Our study shows that females were more likely than males to register as potential deceased organ donors, echoing previous findings that females are often the majority in non-commercial living donations due to their generally more emotional nature, though they are less represented as deceased donors [44,45]. Age also correlates interestingly with organ donation registration, inversely so in our study. According to the Saudi General Authority for Statistics, youth (15–34 years) make up 36.70% of the population as of 2019. This observation is in line with multiple previous studies [29,46,47]. On the contrary, individuals over 45 years old were more aware of Islamic Fatwas approving brain death and organ donation. This age-related disparity could be due to younger individuals being more informed about current issues and the shortage in the donor pool, while older individuals may have emotional concerns regarding body manipulation for organ harvesting. Several studies have highlighted the importance of religion across different age groups, which remains a consistent influence for both young and old [36]. Contrarily, a separate study found that Muslims with more favorable attitudes toward organ donation were typically younger, more open to learning about organ donation, and seemingly more emotionally connected to the topic [38].

HCWs were more aware of the Islamic Fatwas and the role of the SCOT, which reflected in a higher propensity to register as potential organ donors compared to others, resonating with the behavior of global healthcare professionals [29,48].

Participants who supported organ donation registration at the time of issuing driving licenses were significantly more likely to register as potential deceased organ donors. They also held stronger beliefs in brain death and demonstrated greater awareness of the related Fatwas. Moreover, they exhibited an increased willingness to donate organs to friends. Future research should consider methods to enhance public electronic registration as potential donors, potentially accelerating organ allocation if integrated with machine learning (ML) technology within a personalized healthcare system [49,50].

The belief in public and social media campaigns as effective means to promote organ donation was associated with a higher likelihood of participants registering for organ donation. On the other hand, a lack of awareness of Fatwas was linked to a reluctance to donate. However, exposure to a brief educational intervention clarifying Islamic endorsements of organ donation notably increased participants’ willingness to consider organ donation. Engaging Muslim religious leaders in this educational process has been suggested as an effective approach to improving attitudes towards organ donation within Muslim communities. In a display of humanitarian leadership, King Salman and Crown Prince Mohammed bin Salman registered in the Kingdom’s organ donor program in 2021. Other Muslim-majority countries have adopted varied methods to boost living and deceased kidney transplantation rates, including systemic changes and educational campaigns to increase awareness of organ donation in intensive care units in Saudi Arabia, as well as introducing regulated compensation for living-unrelated organ donors in Iran [51,52,53]. A recent survey emphasized that enhancing public awareness about the organ donation process, especially regarding its religious acceptability, could promote organ donation [54]. As shown in the study by Hafzalah et al., providing focused and personalized education can significantly alter perceptions and attitudes [55].

### Study Strengths and Limitations

Our study stands as the first regional investigation to describe the actual willingness to donate organs through the national registration health passport app (Tawakkalna). Despite this novel approach, the study is not without its limitations. It relied on self-reported data, which introduces the potential for social desirability bias. Additionally, the use of social media for participant recruitment may lead to selection bias, potentially affecting the generalizability of our findings to the broader Saudi population, even though the platforms utilized are highly prevalent in KSA. Future research should focus on using a variety of recruitment strategies to obtain a more representative sample.

## 5. Conclusions

The study uncovers a significant discrepancy between the public’s positive perception of organ donation and the actual number of individuals registered as potential deceased donors in Saudi Arabia. We aim to bolster transplant rates, which will lead to enhanced patient outcomes and an improved quality of life. There is a critical need for more targeted public awareness initiatives, particularly those highlighting religious views on organ donation, to narrow this gap. The policy of linking organ donation registration with the issuance of driver’s licenses has garnered substantial backing within our study group and may serve as a promising method to boost registration numbers. To gain a deeper understanding, we advocate for future studies that employ diverse recruitment strategies and assess longitudinal data.

## Figures and Tables

**Table 1:** Descriptive analysis of people’s sociodemographic characteristics. N = 2329.

Characteristic	Frequency	Percentage
**Sex**		
Female	1173	50.4
Male	1156	49.6
**Age group**		
18–24	587	25.2
25–34	621	26.7
35–44	587	25.2
45–54	368	15.8
55–64	166	7.1
**Nationality**		
Non-Saudi	289	12.4
Saudi	2040	87.6
**Educational Level**		
High School	526	22.6
Bachelor’s Degree	1265	54.3
Master’s Degree	242	10.4
Doctorate Degree	296	12.7
**HCW**		
No	1283	55.1
Yes	1195	44.9

**Table 2:** Participants’ perceived organ donation motives and hinderances.

	Frequency	Percentage
**Organ donation registration motives. N = 453**		
Personal or religious belief of helping others and saving lives	412	90.9
Awareness of need for organ donation and its shortage	243	53.6
A desire to leave a positive legacy or make a difference in the world	236	52.7
Recognition of the possibility of receiving an organ transplant in the future if needed	97	21.4
Personal experience with a loved one who required an organ transplant	71	15.7
Other motives	25	5.5
**Organ donation registration hinderances. N = 1726**		
Not convinced	651	37.7
Did not have enough time to register	391	22.7
Unaware about registration	384	22.2
Other reasons	300	17.4
**Persuasive strategies for organ donation registration. N = 2047**		
Raising community awareness of organ donation Fatwa	1361	66.5
Conducting public awareness campaigns	1292	63.1
Integrating organ donation theme in school and university curriculums	1155	56.4
Conducting social media campaigns	1135	55.4

**Table 3:** Multivariable binary logistic regression analysis of participants’ characteristics associated with organ donation registration.

	Multivariate Adjusted (OR)	OR 95% C.I.		*p*-Value
		Lower	Upper	
**Sex**	0.658	0.518	0.836	0.001
**Age ≤ 45 years**	0.894	0.807	0.990	0.031
**Saudi**	2.334	1.592	3.422	<0.001
**HCW**	1.176	1.034	1.337	0.013
**Educational level**	1.214	1.061	1.390	0.005
**Has a relative awaiting organ transplant**	1.460	1.099	1.939	0.009
**Belief in brain death**	0.828	0.517	1.327	0.433
**Brain death Fatwa awareness**	1.256	0.954	1.654	0.105
**Organ donation Fatwa awareness**	1.810	1.372	2.388	<0.001
**Willingness to donate organs to family member**	1.069	0.677	1.688	0.774
**Willingness to donate organs to friend**	2.493	1.952	3.185	<0.001
**Linking organ donation registration decision with driving license**	2.208	1.731	2.818	<0.001
**Previous blood donation**	1.438	1.121	1.844	0.004
**Awareness of SCOT role**	0.695	0.540	0.894	0.005
**Public campaign’s role**	1.290	1.005	1.656	0.046
**Social media campaign’s role**	1.376	1.078	1.757	0.010
**Constant**	0.021			<0.001

Dependent variable: registration as potential deceased organ donor in Tawakkalna app.

**Table 4:** Multivariable binary logistic regression analysis of participants’ awareness of brain death Islamic Fatwa.

	Multivariate Adjusted (OR)	OR 95% C.I.		*p*-Value
		Lower	Upper	
Age ≥ 25 years	1.325	1.219	1.441	<0.001
Saudi	1.492	1.109	2.008	0.008
Educational level	1.088	0.969	1.222	0.154
HCW	1.652	1.482	1.842	<0.001
Has a relative awaiting organ transplant	1.506	1.166	1.944	0.002
Brain death belief	1.883	1.229	2.885	0.004
Willingness to donate organs to family member	1.328	0.971	1.817	0.076
Organ donation registration	1.697	1.353	2.129	<0.001
Linking organ donation registration decision with driving license	1.260	1.017	1.562	0.035
Awareness of SCOT role	2.564	2.027	3.245	<0.001
Perception of organ donation importance	0.792	0.697	0.899	<0.001
Constant	0.029			<0.001

Dependent variable: aware of the brain death Fatwa No/Yes.

**Table 5:** Multivariable binary logistic regression analysis of participants’ awareness of organ donation Fatwa.

	Multivariate Adjusted (OR)	OR 95% C.I.		*p*-Value
		Lower	Upper	
Age ≥ 25 years	1.158	1.071	1.251	<0.001
Saudi	1.418	1.073	1.875	0.014
Believes in brain death	1.181	0.831	1.680	0.354
Willingness to donate organs to family member	1.172	0.868	1.580	0.300
HCW	1.282	1.156	1.421	<0.001
Has a relative awaiting organ transplant	1.336	1.038	1.719	0.024
Organ donation registration	1.916	1.520	2.415	<0.001
Linking organ donation registration decision with driving license	1.329	1.089	1.621	0.005
SCOT role awareness	2.535	2.065	3.113	<0.001
Perception of organ donation importance	0.776	0.694	0.867	<0.001
Previous blood donation	1.265	1.027	1.557	0.027
Constant	0.209			<0.001

Dependent variable: aware of the organ donation religious Fatwa No/Yes.

**Table 6:** Multivariable binary logistic regression analysis of participants’ willingness to donate organs to a friend.

	Multivariate Adjusted (OR)	OR 95% C.I.		*p*-Value
		Lower	Upper	
Sex	1.135	0.949	1.358	0.165
Age ≥ 35 years	0.920	0.852	0.994	0.034
Believes in brain death	1.385	0.961	1.997	0.081
Brain death Fatwa awareness	1.064	0.840	1.348	0.607
Organ donation Fatwa awareness	1.111	0.888	1.390	0.359
Educational Level Master’s degree or higher education	0.769	0.691	0.856	<0.001
Willingness to donate organs to family member	16.790	10.360	27.212	<0.001
Organ donation registration	2.457	1.933	3.125	<0.001
Linking organ donation registration decision with driving license	1.292	1.076	1.550	0.006
Constant	0.110			<0.001

Dependent variable: participants’ willingness to donate organs to friends.

**Table 7:** Multivariable binary logistic regression analysis of participants’ awareness of SCOT role.

	Multivariate Adjusted (OR)	OR 95% CI		*p*-Value
		Lower	Upper	
Age 25 years or above	1.161	1.060	1.273	0.001
Saudi	1.836	1.341	2.514	<0.001
Educational level	1.156	1.012	1.321	0.032
HCW	1.243	1.103	1.400	<0.001
Brain death Fatwa awareness	1.710	1.299	2.251	<0.001
Organ donation Fatwa awareness	1.959	1.538	2.494	<0.001
Perception of organ donation importance	3.101	2.718	3.537	<0.001
Social media campaigns’ role	1.336	1.082	1.652	0.007
Constant	0.013			<0.001

Dependent variable: awareness about the SCOT role.

**Figure A1:**
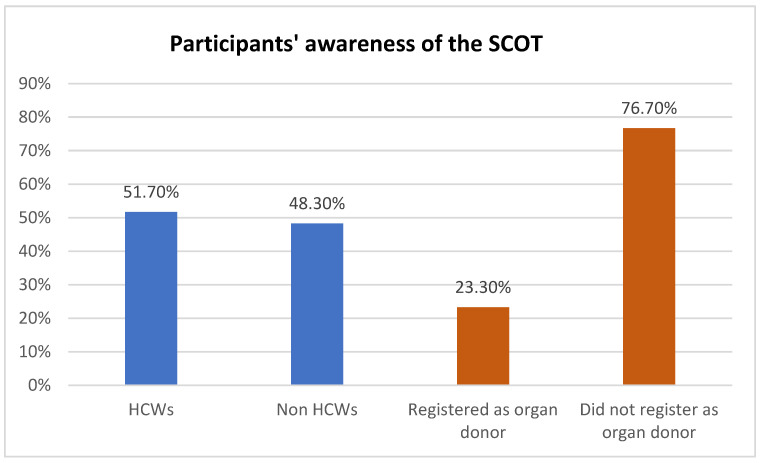
Participants’ awareness of the SCOT (Saudi National Center for Organ Transplant).

## Data Availability

The deidentified participant data collected for this study will be made available upon reasonable request from the corresponding author, in agreement with the IRB-provided signed data sharing agreement.

## Abbreviations

HCWs	Healthcare workers
SCOT	Saudi National Center for Organ Transplant
Tawakkalna	The mobile application launched by the Saudi Ministry of Health in response to the COVID-19 pandemic. This health passport is utilized for registering and verifying the health status and vaccination records of individuals, facilitating the monitoring and management of public health, and includes optional willingness to donate organs for individuals.

## Appendix A

**Table A1:** Descriptive analysis of participant’s attitudes toward organ donation. N = 2329.

	Frequency	Percentage
**Participants’ history of blood donation**		
No	1355	58.2
Yes	974	41.8
**Awareness of SCOT**		
No	871	37.4
Yes	1458	62.6
**Participants’ support of linking driving license issuance with organ donation decision**		
No	1062	45.6
Yes	1267	54.4
**Do you have a relative who is suffering from a disease and needs an organ donation?**		
No	1991	85.5
Yes	338	14.5
**If you registered as a potential organ donor, what organs would you donate?**		
All organs	406	89.2
Kidneys	43	9.5
Liver	35	7.7
Heart	19	4.2
Bone marrow	10	2.2
Lungs	18	4
Cornea	10	2.2
Pancreas	17	3.7

Participants’ age, sex, or education level did not have any significant correlation with their belief in brain death. The strongest predictor of their belief in brain death was their awareness of the Islamic brain death Fatwa (2.038 times more, *p*-value = 0.004), while their awareness of organ donation Fatwa did not have any correlation. Those who had strong beliefs about linking driving license issuance and organ donation decisions and of social media campaigns’ role in encouraging organ donation had strong beliefs about brain death (OR = 1.706, *p*-value = 0.005, OR = 1.669, *p*-value = 0.009), respectively. HCWs also had a significant belief in brain death (OR = 1.508, *p*-value = 0.001). Registration as a potential organ donor was not a significant predictor of belief in brain death (Table A2).

**Table A2:** Multivariable binary logistic regression analysis of participants’ belief in brain death.

	Multivariate Adjusted Odds Ratio (OR)	OR 95% C.I.		*p*-Value
		Lower	Upper	
Age	0.875	0.763	1.005	0.058
Sex	1.090	0.752	1.580	0.648
Educational level	1.179	0.933	1.489	0.168
HCW	1.508	1.194	1.904	0.001
Brain death Fatwa awareness	2.038	1.262	3.290	0.004
Organ donation Fatwa awareness	0.865	0.585	1.280	0.469
Donate organs for a friend	1.419	0.982	2.050	0.062
Registered as potential deceased organ donor	0.800	0.501	1.276	0.348
Organ donation registration decision upon issuing driving license	1.706	1.176	2.473	0.005
Believes social media campaigns would encourage organ donation	1.669	1.139	2.447	0.009
Constant	4.928			<0.001

Dependent variable: do you believe in clinical brain death? (No/Yes).

Participants’ predictors to donate organs to family members have shown a very strong correlation with their willingness to donate organs to friends (16.70 times more, *p*-value < 0.001). The second most significant predictor was their belief in organ donation Fatwa awareness as a motive to enhance society organ donation (1.632 times more, *p*-value = 0.003). Those who previously donated blood were also significantly more likely to donate organs to relatives. Participants who were 45 years of age or older and males were significantly less likely to donate organs to relatives (OR = 0.851, *p*-value = 0.007, OR = 0.674, *p*-value = 0.015), respectively. While those who were triggered to search for organ donation topics after the survey were more willing to donate organs to family members (OR 1.498, *p*-value = 0.010).

**Table A3:** Multivariable binary logistic regression analysis of participants’ willingness to donate organs for family members.

	Multivariate Adjusted Odds Ratio (OR)	OR 95% C.I.		*p*-Value
		Lower	Upper	
Sex Male	0.674	0.490	0.927	0.015
Age ≥45 years	0.851	0.757	0.956	0.007
Donate organ for a friend	16.693	10.332	26.970	<0.001
History of blood donation	1.602	1.132	2.267	0.008
Awareness of SCOT role	0.794	0.576	1.094	0.158
Organ donation Fatwa awareness	1.632	1.187	2.244	0.003
Organ donation search	1.498	1.104	2.033	0.010
Constant	4.646			<0.001

Dependent variable: participants’ willingness to donate organs to relatives.
